# The microRNA landscape and regulatory network in *Clonorchis sinensis*-infected hepatocellular carcinoma: implications for tumor progression

**DOI:** 10.1186/s13071-025-06689-z

**Published:** 2025-02-21

**Authors:** Caibiao Wei, Junxian Chen, Taijun Huang, Lingling Zhou, Yulong Xu, Qiumei Lin, Yuling Qin, Zeli Tang, Weilong Yang, Min Fang

**Affiliations:** 1 Department of Clinical Laboratory, Guangxi Medical University Cancer Hospital, Nanning, 530021 China; 2 Department of Cell Biology and Genetics, School of Basic Medical Sciences, Guangxi Medical University, Nanning, 530021 China; 3 Guangzhou Women and Children’s Medical Center, Guangzhou Medical University, Guangzhou, China; 4 Engineering Research Center for Tissue and Organ Injury and Repair Medicine, Guangxi Medical University Cancer Hospital, Nanning, 530021 China

**Keywords:** *Clonorchis sinensis*, Hepatocellular carcinoma, microRNAs, Progression

## Abstract

**Background:**

Hepatocellular carcinoma (HCC) is a leading cause of cancer mortality globally, and its progression is associated with various factors, including parasitic infections such as *Clonorchis sinensis* (*C. sinensis*). Although *C. sinensis* infection has been implicated in HCC, the molecular mechanisms, particularly the role of microRNAs (miRNAs), remain poorly understood. This study aims to fill this gap by investigating the miRNA expression profiles in *C. sinensis*^+^ and *C. sinensis*^−^ HCC tissues.

**Method:**

We performed miRNA sequencing on HCC tissues from *C. sinensis*^+^ and *C. sinensis*^−^ patients, followed by bioinformatics analyses to identify differentially expressed miRNAs (DEMs) and their target genes. Gene Ontology (GO) enrichment analysis was conducted to explore relevant biological processes, while a competitive endogenous RNA (ceRNA) network was constructed to investigate the interactions among miRNAs, long noncoding RNAs (lncRNAs), and messenger RNAs (mRNAs). Additionally, we performed survival analysis using Gene Expression Profiling Interactive Analysis 2 (GEPIA2) based on the The Cancer Genome Atlas-Liver Hepatocellular Carcinoma (TCGA-LIHC) cohort and assessed the clinical relevance of DEMs. Key miRNAs identified from this analysis were further validated through quantitative real‑time polymerase chain reaction (qRT-PCR) assays to confirm their expression in MHCC97H.

**Results:**

Our research identified significant miRNA dysregulation in *C. sinensis*^+^ HCC tumors compared with *C. sinensis*^−^ HCC tumors. Notably, miR-143-3p, miR-10a-5p, and miR-100-5p were upregulated in *C. sinensis*^+^ HCC, contributing to immune responses and tumor progression, while let-7 family members and miR-221-3p were downregulated, affecting metabolic pathways. GO enrichment analysis highlighted the involvement of developmental processes, immune system regulation, and metabolic reprogramming in *C. sinensis*^+^ HCC. The construction of a ceRNA network revealed key interactions between miRNAs, lncRNAs, and mRNAs in *C. sinensis*^+^ HCC, suggesting regulatory mechanisms that could be potential therapeutic targets. Additionally, validation through qRT-PCR confirmed these findings, highlighting miRNA dysregulation as a critical factor in *C. sinensis*^+^ HCC progression.

**Conclusions:**

This study provides novel insights into the role of miRNAs in *C. sinensis*-infected HCC progression. The findings highlight the critical role of miRNA dysregulation in the progression of *C. sinensis*-associated HCC, emphasizing the potential for therapeutic interventions targeting these molecular alterations in affected patients.

**Graphical Abstract:**

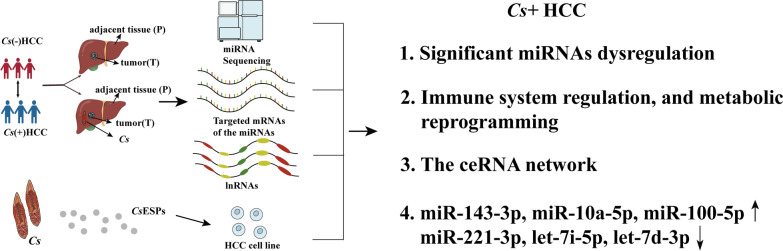

**Supplementary Information:**

The online version contains supplementary material available at 10.1186/s13071-025-06689-z.

## Background

Hepatocellular carcinoma (HCC) is among the most prevalent and lethal cancers worldwide [1]. There are approximately 870,000 new cases and 760,000 deaths annually [2]. Despite significant advances in research and therapeutic strategies, the prognosis for HCC remains poor [3]. The development of HCC is a complex, multistep process involving multiple genetic and epigenetic alterations [4]. Liver fibrosis, chronic viral infections, nonalcoholic fatty liver disease (NAFLD), type 2 diabetes, and obesity are significant risk factors contributing to the development, progression, and poor prognosis of HCC [5–7]. Recently, increasing evidence suggests that certain parasitic infections, such as *Clonorchis sinensis* (*C. sinensis*), are also linked to adverse outcomes in HCC [8–11].

*C. sinensis* is one of the most severe foodborne parasites, with the highest prevalence in China, affecting approximately 13 million people [12]. Datao Lin et al. showed that, on the basis of data from a tertiary hospital in Guangdong Province, the direct medical costs per case of Chinese testicular schistosomiasis were estimated to be approximately RMB 7907.2, while the total economic burden of liver and gallbladder diseases caused by the infection amounted to RMB 1.6 billion, including indirect costs such as medical expenses and labor losses [13]. Eating raw freshwater fish containing *C. sinensis* metacercariae is high-risk behavior to get infected *C. sinensis*, which can cause hepatobiliary diseases in the epidemic areas, such as clonorchiasis, liver cirrhosis, and cholangiocarcinoma, which all significantly raise the risk of developing liver cancer [14]. Recent studies indicate that *C. sinensis* is linked to poor prognosis in HCC through various mechanisms, including inducing chronic inflammation, altering cellular signaling pathways such as TGF-β, and affecting immune responses, such as immune evasion and immune suppression [15, 16]. Additionally, recent research highlights its role in enhancing stemness and further contributing to HCC progression [8]. Previous studies have demonstrated distinct expression and regulatory patterns of genes across different developmental diseases of *C. sinensis* [17–19]. For instance, Yangyuan Qiu conducted whole-transcriptome sequencing across different developmental stages of *C. sinensis* in rabbits [19]. Tingzheng Zhan et al. using multi-omics approaches revealed the molecular mechanisms underlying the interaction between *C. sinensis* and mouse liver [18]. These findings indicate that each developmental stage and different tissues are associated with distinct biological properties and pathogenic features, highlighting the intricate *C. sinensis* regulatory mechanisms. However, these studies are primarily based on multi-omics approaches using animal models. To date, there are no microRNAs (miRNAs) sequencing data related to hepatocellular carcinoma patients associated with *C. sinensis*.

miRNAs are a class of endogenous short single-stranded noncoding RNA molecules, typically 18–25 nucleotides in length [20]. They regulate gene expression at the posttranscriptional level by binding to complementary sequences on target messenger RNAs (mRNAs) molecules [21, 22]. This binding can lead to degradation or translational repression of the mRNAs, thereby regulating the expression of genes involved in important cellular processes [21, 23]. In addition, miRNAs interact with long noncoding RNAs (lncRNAs) to regulate physiological processes [24, 25]. Aberrant miRNAs expression is associated with HCC development and progression [26, 27]. Determining the miRNAs expression profile provides valuable insights into the intricate biology of *C. sinensis*-infected HCC, thereby offering information on potential targets for understanding its pathogenicity and for developing effective interventions.

This study is the first to reveal the dynamic miRNA landscape in *C. sinensis*-infected HCC patients. By analyzing the expression profiles of miRNAs in *C. sinensis*^+^ and *C. sinensis*^−^ HCC tissues, we identified differentially expressed miRNAs, elucidated their biological functions through Gene Ontology (GO) enrichment analysis, and constructed competitive endogenous RNA (ceRNA) networks. These findings provide critical insights into how *C. sinensis* infection influences miRNA regulatory pathways, contributing to immune evasion, metabolic reprogramming, and tumor progression. Furthermore, the results highlight potential therapeutic targets and distinct prognostic subclasses, offering novel opportunities for improving the clinical management of *C. sinensis*-infected HCC.

## Methods

### Human samples

Human liver tissue samples were obtained from treatment-naive patients with HCC who had no other malignancies and underwent surgical resection at the Department of Hepatobiliary Surgery, Affiliated Cancer Hospital of Guangxi Medical University (Nanning, China). All patients were informed about the procedures and provided written informed consent. HCC tissues with typical macroscopic features were collected from tumor nodules and confirmed by hematoxylin and eosin (H&E) staining. Adjacent noncancerous tissues were obtained from 5 cm above the tumor border. The study protocol was approved by the Ethics Committee of the Affiliated Cancer Hospital of Guangxi Medical University (LW2024125). The inclusion criteria were as follows: (1) hepatectomy with a postoperative pathological diagnosis of HCC; (2) hepatectomy as the first treatment with no history of other malignant tumors; (3) all patients underwent testing for *C. sinensis* infection at the time of initial diagnosis; (4) *C. sinensis* ^+^ patients did not receive antiparasitic treatment prior to surgical resection. The exclusion criteria were as follows: patients co-infected with hepatitis A, C, D, or E, human immunodeficiency virus (HIV), or other parasites; those with autoimmune diseases, severe heart conditions, or diabetes; and pregnant women were excluded. We diagnosed *C. sinensis* infection on the basis of the following criteria. Meeting any one of these criteria was sufficient to establish the diagnosis: (1) preoperative fecal examination showing that the presence of *C. sinensis* eggs; (2) intraoperative or postoperative pathological examination revealing the presence of adult *C. sinensis* in the liver or gallbladder.

### MicroRNA sequencing (miRNA-seq)

During the RNA extraction process, an equal volume of chloroform (1/5 of the total volume) was added to the sample, followed by vigorous agitation and a 2–3 min incubation at room temperature to facilitate phase separation, thereby isolating the RNA-containing upper aqueous phase. Subsequently, the aqueous phase was carefully transferred to a new centrifuge tube, where an equal volume of isopropanol was added, mixed, and incubated at room temperature for 10 min to promote RNA precipitation. After centrifugation, the supernatant was discarded, and the RNA pellet was washed with 75% ethanol before final centrifugation and removal of the supernatant. The pellet was then air-dried at room temperature and subsequently resuspended in RNase-free water (DEPC-treated water), with a portion reserved for analysis and the remaining sample stored at −80 °C for long-term preservation. Total RNA was used for library preparation. Briefly, 3′ and 5′ adapters were ligated to the 3′ and 5′ ends of the small RNAs to facilitate their subsequent conversion into cDNA and sequencing. These adapters are designed to specifically capture and stabilize miRNAs, ensuring accurate and efficient sequencing of the small RNA population. First-strand cDNA was synthesized using reverse transcription primers specifically designed to anneal to the miRNAs. The double-stranded cDNA library was then generated through PCR amplification using primers that amplify the cDNA with the ligated adapters. After purification and size selection, libraries with insert sizes ranging from 18 to 40 bp were prepared for single-end 50 bp Illumina sequencing.

### Collection and preparation of *Cs*-produced excretory/secretory products (*Cs*ESPs)

To collect *Cs*ESPs, we harvested *C. sinensis* metacercariae from naturally infected freshwater fish (*Pseudorasbora parva*) in Hengxian County, Guangxi Zhuang Autonomous Region, China. The fish were processed by removing the head, tail, gills, fins, and internal organs, followed by deboning and cutting into pieces. The fish meat was then ground or chopped and digested overnight at 37 °C in a 0.8% pepsin solution containing 0.2% HCl. The digested mixture was filtered through a 60–80 mesh sieve, and live metacercariae were isolated from the sediment under a microscope, then stored in phosphate-buffered saline (PBS) at 4 °C. Adult *C. sinensis* worms were collected from the bile ducts of infected Sprague–Dawley (SD) rats. The worms were cultured with medium changes every 6 or 12 h. After 48 h of culture, the medium was pooled and centrifuged at 12,000 rpm for 30 min at 4 °C to collect the supernatant. The supernatant was dialyzed in PBS to remove small molecules, and then concentrated using either sucrose or lyophilized, depending on the intended use. The molecular weight and concentration of the sample were measured, and it was stored at −80 °C for future use. Before use, the sample was filtered through a 0.22 µm ultrafiltration membrane to ensure sterility.

### Cell culture

Human HCC cell line MHCC97H was obtained from the Type Culture Collection of the Chinese Academy of Sciences (Shanghai, China). MHCC97H cells were treated with 50 μg/ml *Cs*ESP or PBS for 48 h. These cells were cultured in DMEM medium containing 10% fetal bovine serum (Wisent, Canada) at 37 °C in a humidified 5% CO_2_ atmosphere.

### Quantitative real-time polymerase chain reaction (qRT-PCR)

Total RNA was extracted from the MHCC97H using Trizol reagent (Invitrogen, America). Subsequently, 3.75 μg of RNA was reverse transcribed into cDNA using the Reverse Transcription Master Kit (Takara, Japan) according to the manufacturer’s instructions. qRT-PCR was performed using the qTOWER3 Fluorescence Quantitative PCR Instrument Real-time Fluorescence Quantitative PCR System (Jena, Germany), using TB Green Premix Ex Taq II FAST qPCR (2X) (Takara, Japan). The reaction was first incubated at 95 °C for 30 s, followed by 39 cycles of 95 °C for 5 s and 60 °C for 30 s. Relative gene expression levels were normalized to the expression of U6 (Takara, Japan) miRNAs and calculated using the 2^−ΔΔCt^ method. The Ct value, or cycle threshold value, is determined during qPCR by detecting the cycle at which the fluorescence generated by the target DNA amplification exceeds a predefined threshold level. The experiments were repeated three times to ensure the consistency and reliability of the results. The bulge-loop RT primer and qPCR primers specific for hsa-miR-143-3a, hsa-miR-10a-5p, hsa-miR-100-5p, hsa-let-7i-5p, hsa-let-7d-3p, and hsa-miR-221-3p were designed and synthesized by sangonbiotech (Shang Hai, China). Supplementary Table S1 lists the primers used in this study.

### Analysis of miRNA-Seq and RNA-Seq data

The low-quality reads and adapters from raw sequencing reads were removed by Trim Galore (version 0.6.10), and the clean data were obtained. Clean data were aligned to the hg38 reference genome using Hisat2 (v. 2.2.1) with default parameters. The GFF3 file of miRNAs was downloaded from miRBase (https://www.mirbase.org/). featureCounts (v.2.0.6) was used to compute the miRNAs expression matrix. We used the R package ‘DEseq2’ (v.1.44.0) to normalize gene expression and detect differentially expressed genes (|fold change|> 2 and *P* < 0.05). The information of miRNAs-target interactions was obtained from miRTarBase (https://mirtarbase.cuhk.edu.cn/~miRTarBase/miRTarBase_2022/php/index.php).

### GO enrichment analysis

We utilized the Metascape online tool (https://metascape.org/gp/index.html#/main/step1) to perform GO enrichment analysis with default parameters. This allowed us to explore the biological processes, molecular functions, and cellular components enriched in our target genes and transcription factors (TFs).

### Survival analysis

We analyzed the miRNAs data obtained from our sequencing to identify mRNAs expression genes and utilized these data to screen for mRNAs associated with survival prognosis in HCC patients. Subsequently, we performed survival analysis using the Gene Expression Profiling Interactive Analysis 2 (GEPIA2) website (http://gepia2.cancer-pku.cn/#survival), based on the The Cancer Genome Atlas—Liver Hepatocellular Carcinoma (TCGA-LIHC) cohort with default parameters. A *P*-value of less than 0.05 indicates that the mRNAs are associated with the survival prognosis of HCC patients.

### LncRNA–miRNA–mRNA network build

**Fig. 1:**
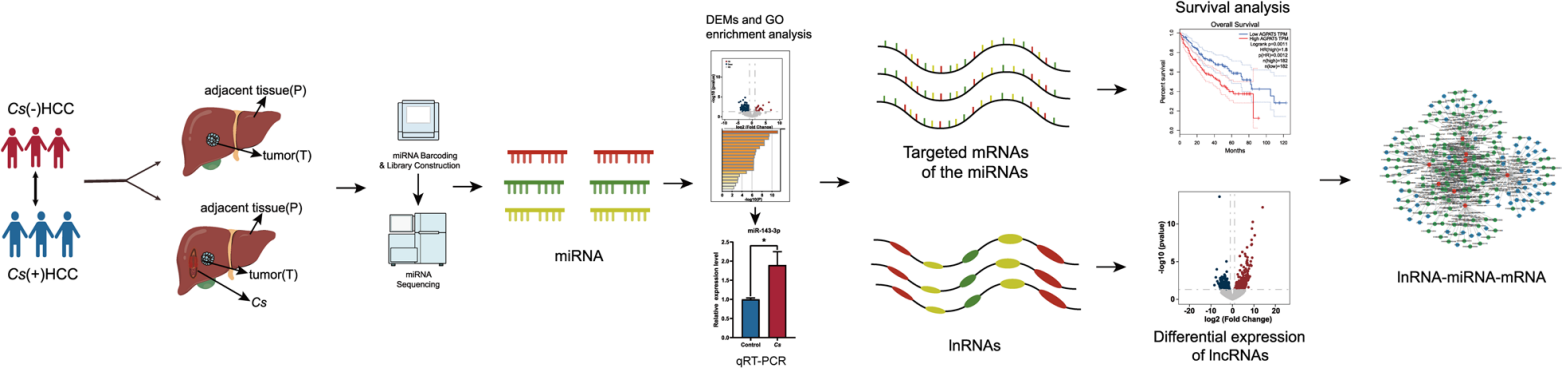
Flowchart of construction of ceRNA network

LncRNA target genes were predicted by mircode (http://www.mircode.org/), and miRNA target genes were predicted by miRTarBase (https://mirtarbase.cuhk.edu.cn/~miRTarBase/miRTarBase_2022/php/index.php). Finally, different expression lncRNAs, miRNAs, and mRNAs were used to build the lncRNA–miRNA–mRNA network, and it was exhibited by cytoscape (v. 3.7.2). The flowchart shows the entire process from selecting differentially expressed miRNAs, mRNAs, and lncRNAs to constructing the ceRNA network, as shown in Fig. 1.

### Statistical analysis

Statistical analysis was conducted by using R software (v.4.3.0) and GraphPad Prism 8.0.2 for Windows. The correlation between variables was evaluated by Pearson’s test, and the independent samples were subjected to a *t*-test or analysis of variance (ANOVA) to investigate group differences. The statistical significance was set at *P* < 0.05.

## Results

### The quality of miRNA data in ***C. sinensis***^+^ and ***C. sinensis***^−^ HCC

**Fig. 2:**
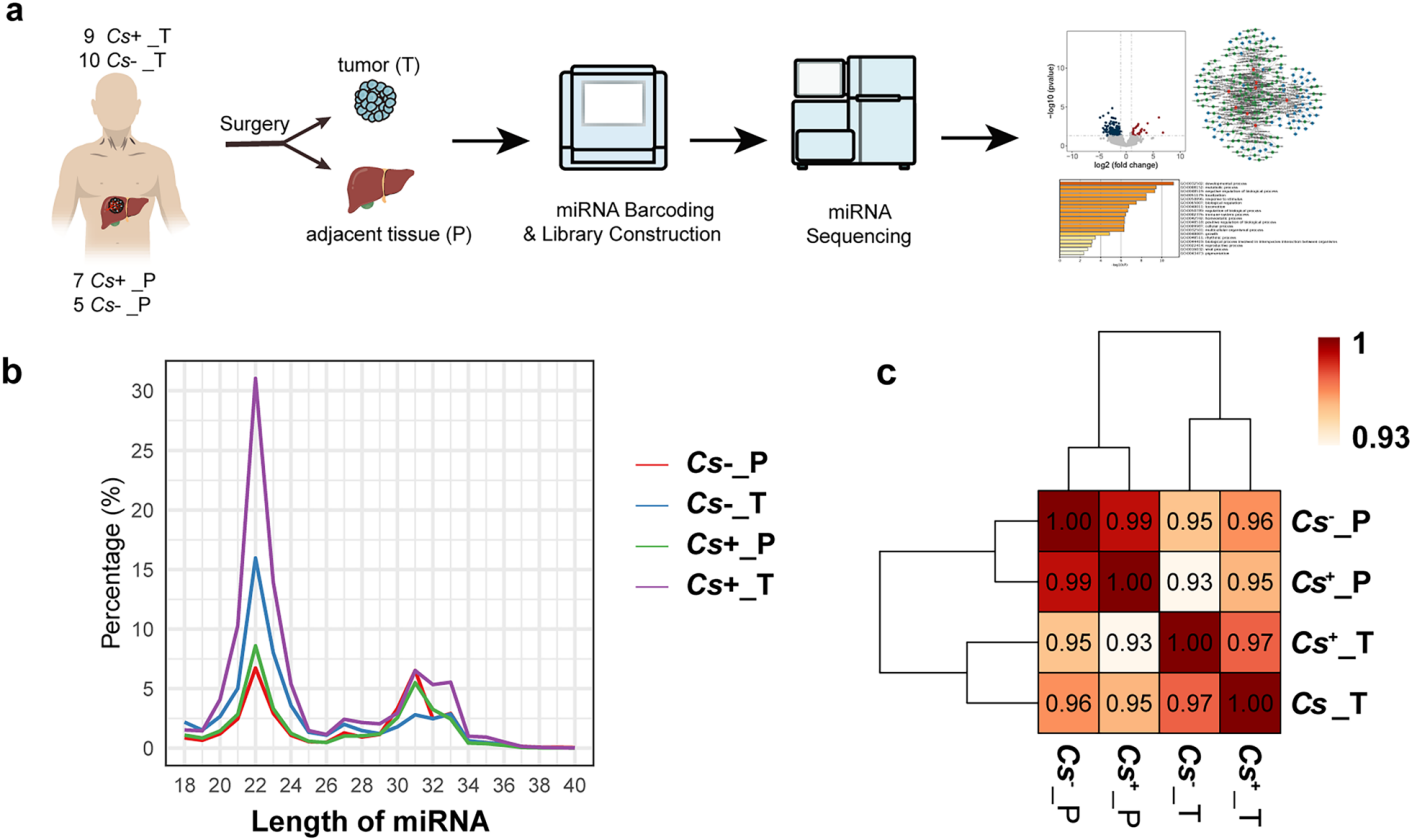
Flow diagram of enrolled participants and evaluation process of *C. sinensis*^+^ and *C. sinensis*^−^ HCC. **a** Experimental strategy for miRNA assay and integration analysis of *C. sinensis*^+^ and *C. sinensis*^−^ HCC. **b** Length distribution of miRNAs data in each group of *C. sinensis*^+^ and *C. sinensis*^−^ HCC. **c** Correlation analysis between all groups

To investigate the impact of *C. sinensis* infection on microRNA expression in HCC, an experimental workflow was designed (Fig. 2a). We collected tumor tissue (*C. sinensis*^+^_T) from nine cases and adjacent nontumor tissue (*C. sinensis*^+^_P) from seven cases in *C. sinensis*^+^ HCC patients, as well as corresponding tumor tissue (*C. sinensis*^−^_T) from ten cases and adjacent nontumor tissue (*C. sinensis*^−^_P) from five cases in *C. sinensis* ^−^ HCC patients, and performed miRNA analysis on these samples. Information for all patients is listed in Supplementary Table S2. The graph displays the length distribution of miRNAs data for different groups in *C. sinensis*^+^ and *C. sinensis*^−^ HCC (Fig. 2b). The miRNAs fragments primarily show a peak around the 22-nucleotide length, which is reflected by the spike in percentage for all groups, most notably in the *C. sinensis*^+^_T group. Finally, correlation analysis showed that *C. sinensis*^+^_T and *C. sinensis*^−^_T samples were more similar to each other, as were *C. sinensis*^+^_P and *C. sinensis*^−^_P samples (Fig. 2c). Additionally, all analyses were also performed at the sample level (Supplementary Fig. S1).

### miRNAs expression profile and biological function analysis in ***C. sinensis***^+^ and ***C. sinensis***^−^ HCC

**Fig. 3:**
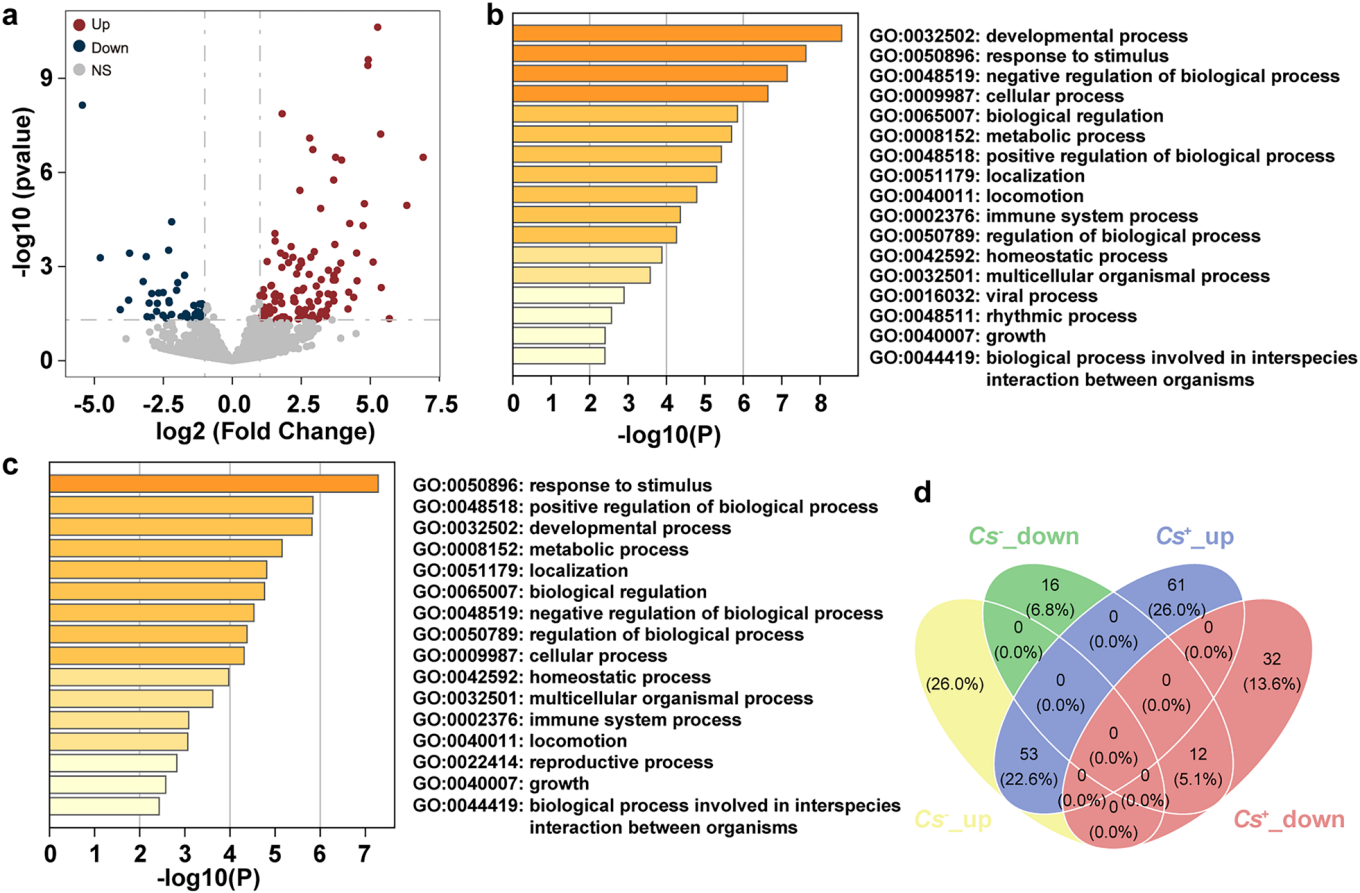
miRNA expression profile and biological function analysis in *C. sinensis*^+^ HCC. **a** The volcano plot of DEMs between *C. sinensis*^+^ HCC tumors and tumor-adjacent tissues. **b**, **c** GO enrichment analysis of biological processes associated with upregulated (**b**) and downregulated (**c**) miRNAs between *C. sinensis*^+^ HCC tumors and tumor-adjacent tissues from patients. **d** The Venn diagram of the relationship between DEMs in *C. sinensis* ^+^ and *C. sinensis*^−^ HCC

We initially assessed the miRNA expression profiles in *C. sinensis*^+^ HCC samples, identifying significantly differentially expressed miRNAs (DEMs). The analysis revealed notable upregulation and downregulation of miRNAs in *C. sinensis*^+^ HCC tumors compared with adjacent tissues. The results showed that, compared with adjacent tissues, 114 miRNAs were significantly upregulated and 44 miRNAs were significantly downregulated in *C. sinensis*^+^ HCC tumors (Supplementary Table S3). These DEMs accounted for 5.96% (158/2652) of the total identified miRNAs (Fig. 3a). To better understand the overall consequences of DEMs in *C. sinensis*^+^ HCC and how these alterations functioned, the biological process was explored with GO enrichment analysis. The GO analysis indicated that the target genes of upregulated miRNAs were primarily enriched in biological processes related to the developmental process, response to stimulus, negative regulation of biological processes, cellular process, and localization (Fig. 3b). Conversely, the target genes of downregulated miRNAs were similarly enriched in biological processes such as response to stimulus, positive regulation of biological processes, developmental processes, metabolic processes, and localization (Fig. 3c). These enrichment results suggest that, although the target genes of both upregulated and downregulated miRNAs are involved in similar biological processes, their expression patterns differ significantly between tumor and adjacent tissues.

Subsequently, the DEMs between tumors and adjacent tissues in *C. sinensis*^−^ HCC were analyzed, and 114 upregulated and 28 DEMs were identified in *C. sinensis*^−^ HCC tumors compared with adjacent tissues (Supplementary Table S4), accounting for 5.40% of the overall DEMs identified (Supplementary Fig. S2a). After GO analysis, the target genes of upregulated DEMs were primarily enriched in processes such as metabolic processes, cellular processes, and the negative regulation of biological processes (Supplementary Fig. S2b). However, the target genes of downregulated DEMs were mainly enriched in processes related to development, response to stimulus, and the positive regulation of biological processes (Supplementary Fig. S2c). Finally, we compared the differences in miRNAs expression between *C. sinensis* ^+^ and *C. sinensis*^−^ HCC tumors and adjacent tissues. Venn diagram analysis revealed substantial differences in miRNAs expression patterns between *C. sinensis* ^+^ and *C. sinensis* ^−^ HCC, particularly in the gene sets associated with upregulated and downregulated miRNAs (Fig. 3d). These findings suggest that *C. sinensis* infection may influence the biological behavior of HCC by altering miRNA expression patterns within the tumor.

### Characterization of miRNAs expression profiles and biological function in HCC following *C. sinensis* infection

**Fig. 4:**
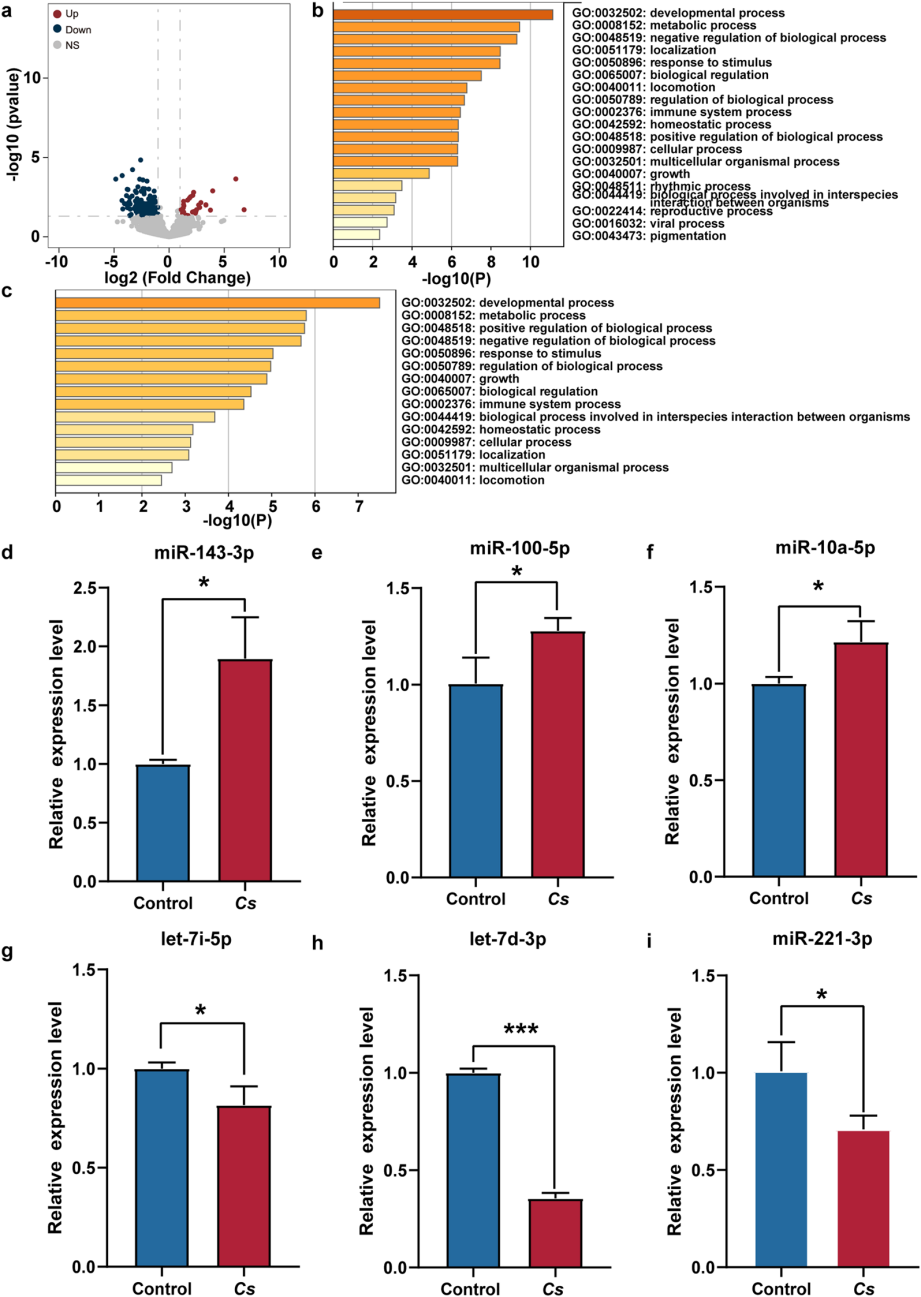
*C. sinensis* infection alters miRNA expression in HCC tumors. **a** The volcano plot of DEMs between *C. sinensis* ^+^ HCC tumors and *C. sinensis *^−^ HCC tumors. **b**, **c** GO enrichment analysis of biological processes associated with upregulated (**b**) and downregulated (**c**) miRNAs between *C. sinensis* ^+^ HCC tumors and *C. sinensis *^−^ HCC tumors. **d**, **i** Expression of (**d**–**f**) hsa-miR-143-3p, hsa-miR-100-5p, and hsa-miR-10a-5p (upregulated), as well as (**g**–**i**) hsa-let-7i-5p, hsa-let-7d-3p, and hsa-miR-221-3p (downregulated), at the cellular level, detected by qRT-PCR in the control and *C. sinensis* groups (*n* = 3/ group). Data are presented as mean ± SD; Student’s *t*-test was used. *P < 0.05, **P < 0.01 and ***P < 0.001

To decipher the regulatory mechanisms underlying the distinct responses to *C. sinensis* infection, the miRNA profiles of *C. sinensis* ^+^ HCC tumors and *C. sinensis* ^−^ HCC tumors were first compared. A total of 32 upregulated and 138 downregulated DEMs were identified in *C. sinensis* ^+^ HCC tumors compared with *C. sinensis*^−^ HCC tumors (Supplementary Table S5), accounting for 6.41% of the total DEMs identified (Fig. 4a). Among the top five upregulated miRNAs with the most significant differential expression, hsa-miR-143-3p and hsa-miR-10a-5p serve as key regulators of immune responses and cell migration, and are associated with tumor progression [28, 29]. Similarly, hsa-miR-100-5p plays a crucial role in cancer progression by regulating apoptosis and cell growth, thereby influencing tumor development [30–32]. Both the upregulated and downregulated miRNAs were selected from the top five most significantly differentially expressed miRNAs, further emphasizing their importance in *C. sinensis* ^+^ HCC. Among the top five most significantly downregulated miRNAs, hsa-let-7i-5p, hsa-let-7d-3p, and hsa-miR-221-3p are involved in the regulation of immune and metabolic pathways and are closely linked to cancer development [33–37]. The results of GO analysis showed that genes targeted by upregulated miRNAs were enriched in processes related to the immune system, locomotion, growth, regulation of biological processes, and positive regulation of biological processes (Fig. 4b). In contrast, genes targeted by downregulated miRNAs were enriched in processes such as developmental process, metabolic process, positive regulation of biological process, immune system, locomotion, etc. (Fig. 4c).

To validate the results of the miRNAs sequencing, we selected the top five upregulated and downregulated miRNAs with the most significant expression differences for qRT-PCR analysis. The results indicated that, compared with *C. sinensis* ^−^ HCC, the expression levels of hsa-miR-143-3p, hsa-miR-10a-5p, and hsa-miR-100-5p were significantly upregulated in *C. sinensis* ^+^ HCC (Fig. 4d–f). Conversely, hsa-let-7i-5p, hsa-let-7d-3p, and hsa-miR-221-3p showed significant downregulation in *C. sinensis* ^+^ HCC compared with *C. sinensis* ^−^ HCC (Fig. 4g–i). Overall, the qRT-PCR results were consistent with our miRNA-seq findings.

To explore whether *C. sinensis* infection also changed the miRNAs of tumor-adjacent tissues, the relevant analyses were performed. We identified 24 increased and 21 decreased DEMs in *C. sinensis* ^+^ HCC tumor-adjacent tissues compared with *C. sinensis* ^−^ HCC tumor-adjacent tissues (Supplementary Table S6), accounting for 2.07% of the overall DEMs identified (Supplementary Fig. S3a). Moreover, the results of GO analysis showed that increased DEMs target genes were enriched for genes involved in the developmental process, response to stimulus, cellular process, locomotion, etc. (Supplementary Fig. S3b), and decreased DEMs target genes were enriched for genes involved in the response to stimulus-metabolic process, multicellular organismal process, etc. (Supplementary Fig. S3c).

### Overall survival (OS) of all DEMs target genes in *C. sinensis*^+^ and *C. sinensis*^−^ HCC tumor patients

**Fig. 5:**
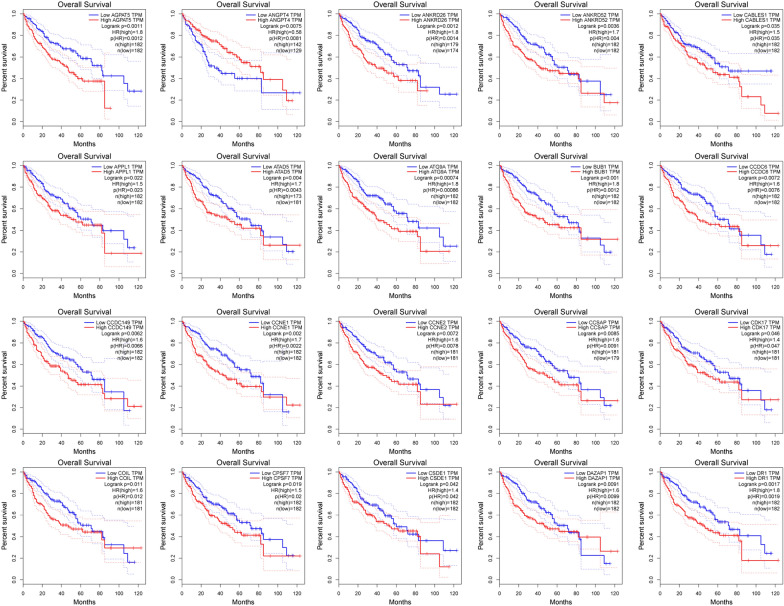
Kaplan–Meier survival curves for mRNAs associated with OS. A prognostic survival analysis of OS in HCC patients, highlighting the impact of 20 selected miRNAs target genes and differentially expressed genes (DEGs) from an initial pool of 228 candidates

On the basis of the regulation of mRNAs by miRNAs [38], we explored the significance of miRNAs in gene expression modulation, particularly how miRNAs influence the expression levels of relevant mRNAs in HCC and how these changes relate to tumor progression and patient prognosis. We identified 766 DEMs’ target genes between *C. sinensis* ^+^ and *C. sinensis* ^−^ HCC patients (Supplementary Table S7). To evaluate the prognostic relevance of these DEMs’ target genes, we performed survival analysis using the GEPIA2 online tool (http://gepia2.cancer-pku.cn/#survival) based on the TCGA-LIHC cohort. We conducted a prognostic analysis of OS in HCC patients on the basis of mRNA expression levels of 766 DEMs’ target genes, identifying 228 genes with significant associations (Supplementary Table S8). Finally, 20 genes were selected for detailed presentation (Fig. 5). For these significant genes, the survival curves of the high-expression and low-expression groups displayed different patterns. High expression of some genes was associated with poorer survival rates (e.g., HR > 1, suggesting a potential oncogenic role), while high expression of other genes was linked to better survival rates (e.g., HR < 1, suggesting a possible tumor-suppressive function). This variability indicates that different genes may play distinct biological roles in HCC, with some genes potentially promoting tumor progression and others potentially involved in tumor suppression.

### ***C. sinensis*** infection induces a different expression profile of lncRNAs in ***C. sinensis***^+^ and ***C. sinensis***^−^ HCC tumors

**Fig. 6:**
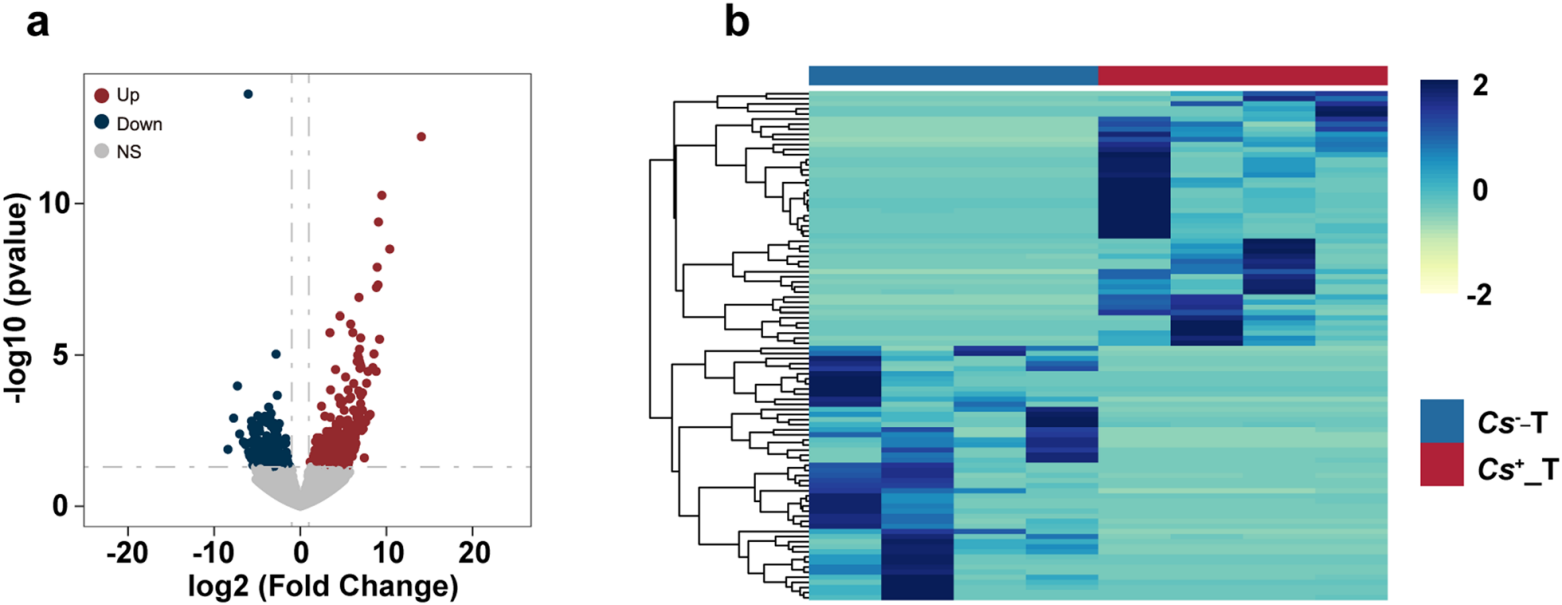
Volcano plot and heatmap of differentially expressed lncRNAs in *C. sinensis*^+^ HCC tumors and *C. sinensis*^−^ HCC tumors. **a** The volcano plot reflects the differential expression of lncRNAs between *C. sinensis*^+^ HCC tumors and *C. sinensis*^−^ HCC tumors. **b** The heatmap shows the top 50 differentially up- and downregulated lncRNAs between *C. sinensis*^+^ and *C. sinensis*^−^ tumors

Studies have shown that lncRNAs can sponge miRNAs, influencing normal physiological processes and regulating mRNA expression, thereby affecting the physiological state of cells [39]. To further explore the relationship between the expression levels of lncRNAs and miRNAs, we recalled our RNA-Seq data for *C. sinensis*^+^ HCC tumors and *C. sinensis* ^−^ HCC tumors from another study by us (GSE276855) and calculated the differentially expressed lncRNAs between *C. sinensis*^+^ HCC tumors and *C. sinensis*^−^ HCC tumors (Fig. 6a). Our analysis revealed 279 lncRNAs with increased expression and 214 lncRNAs with decreased expression in *C. sinensis*^+^ HCC tumor tissues compared with *C. sinensis*^−^ HCC tumor tissues, accounting for 2.46% (493/20057) of the overall lncRNAs identified (Table S9). Notably, ENSG00000240040 emerged as the top-ranked lncRNAs with decreased expression, while ENSG00000253898 ranked first among the lncRNAs with increased expression. The heatmap illustrated the expression levels of lncRNAs between *C. sinensis*^+^ HCC tumors and *C. sinensis*^−^ HCC tumors (Fig. 6b).

### Construction of the lncRNA–miRNA–mRNA network

**Fig. 7:**
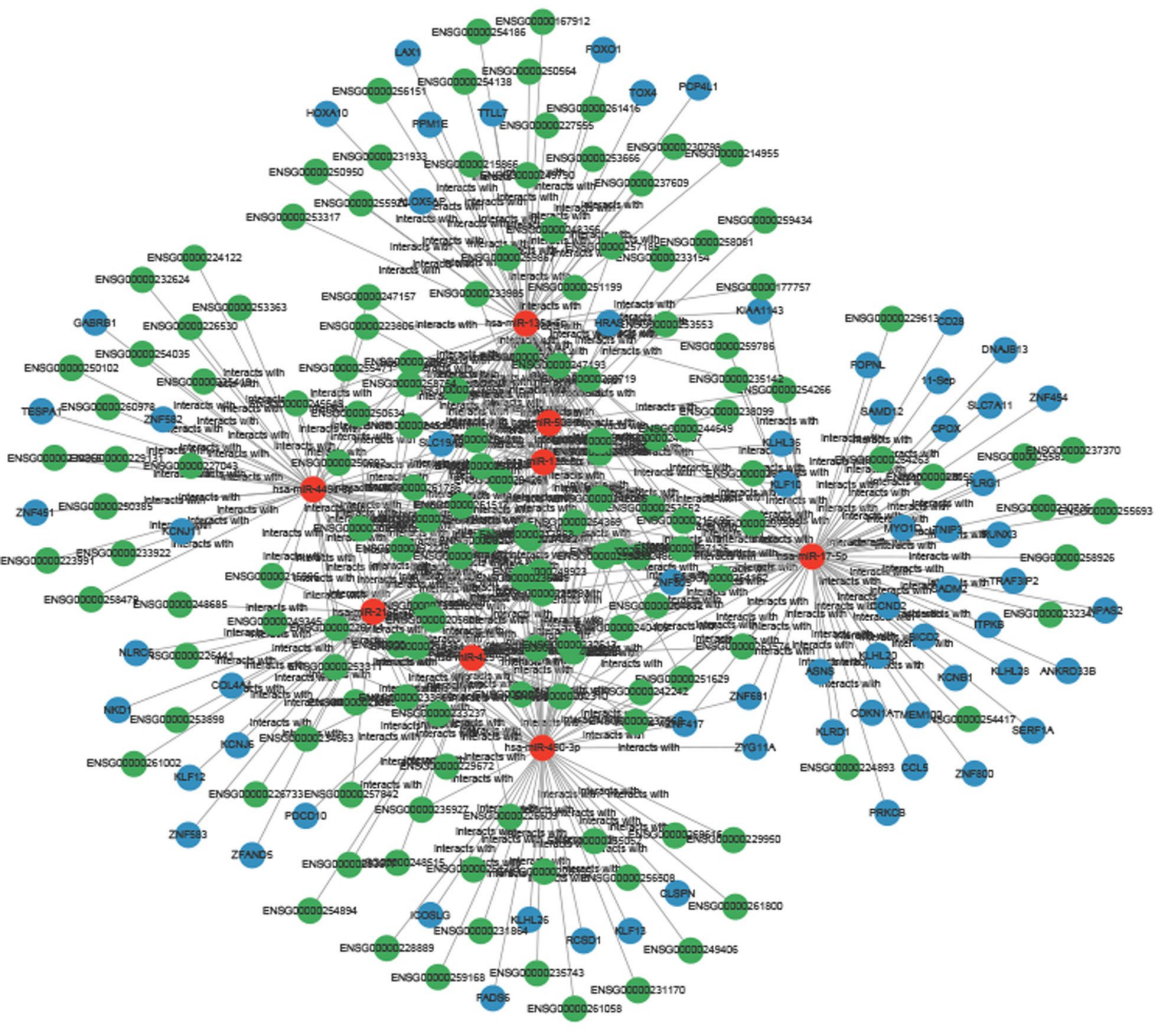
A ceRNA network model that represents the computationally predicted interactions among lncRNAs, miRNAs, and mRNAs in the context of *C. sinensis* infection. The lncRNA nodes are represented by blue circles, the miRNA nodes are represented by red circles, and the mRNA nodes are represented by green circles

miRNAs play a pivotal role in the regulation of gene expression by interacting with mRNAs, thereby influencing transcription and translation processes [38]. Similarly, lncRNAs are critical regulators in various cellular processes, including the cell cycle, differentiation, and apoptosis [40]. In the progression of HCC, miRNAs interact not only with mRNAs but also with lncRNAs, forming a complex network that regulates key biological processes. To further explore the intricate relationships among lncRNAs, miRNAs, and mRNAs in HCC, we constructed a ceRNA network. This network revealed that 149 differentially expressed lncRNAs regulate 8 miRNAs, which, in turn, regulate 66 mRNAs, highlighting the regulatory interactions among these molecules (Supplementary Table S10). A total of 223 nodes and 384 edges were identified (Fig. 7), illustrating the complex interactions among these molecules (Supplementary Tables S10, S11). The centrally located miRNAs (red nodes) are predicted to be major regulators within this network, interacting with multiple mRNAs and lncRNAs. LncRNAs nodes (blue) regulate miRNAs availability through a “sponge effect," influencing their interactions with target mRNAs. mRNAs node (green) function as downstream effector molecules, participating in signaling pathways and cellular processes. Among the identified miRNAs, hsa-miR-139-5p, hsa-miR-425-5p, hsa-miR-490-3p, hsa-miR-449c-5p, hsa-miR-135a-5p, hsa-miR-216b-5p, hsa-miR-17-5p, and hsa-miR-508-3p are likely involved in critical processes such as cell proliferation and metastasis, particularly in the context of *C. sinensis* infection. Although experimental validation was not performed in this study, previous research, including miRNAs analyses in animal models infected with *C. sinensis*, has demonstrated the significant impact of *C. sinensis* infection on liver pathology (e.g., hepatic fibrosis, immune response, and liver damage). These findings provide strong support for the potential role of miRNAs in HCC progression and offer new perspectives for exploring miRNAs function in host responses.

## Discussion

*C. sinensis*, a parasitic zoonosis prevalent in Asia, poses a remarkable disease burden [13]. Recently, more and more research has reported that it is related to poor prognosis in HCC, but the specific mechanisms and mediators are not fully understood [8–11, 41]. Increasing evidence indicates that epigenetic miRNAs alterations play a central role in the development of HCC [42, 43]. In this study, we conducted a comprehensive analysis of miRNAs expression profiles in *C. sinensis* ^+^ and *C. sinensis* ^−^ HCC tumors and adjacent tissues, revealing significant miRNAs dysregulation that highlights the complex interplay between miRNAs, lncRNAs, and mRNAs in HCC pathogenesis. Our study provides novel insights into how *C. sinensis* infection promotes HCC progression via miRNAs dysregulation and ceRNA networks, revealing potential therapeutic targets.

The carcinogenesis of *C. sinensis* encompasses a variety of factors, such as mechanical obstruction, toxic effects of the worms’ ESPs, and immune regulation [44, 45]. Research indicates that, during the progression of infectious diseases, the host’s miRNAs expression profile undergoes significant alterations, which are closely associated with impairments in host biological functions [46]. Through miRNA-seq analysis of *C. sinensis* ^+^ and *C. sinensis* ^−^ HCC tumor samples, we identified several miRNAs that play key roles in regulating immune responses and metabolic pathways. Among them, hsa-miR-143-3p, hsa-miR-10a-5p, hsa-miR-100-5p, and members of the let-7 family (e.g., let-7d-3p, let-7i-5p), hsa-miR-221-3p were highlighted as potential regulatory factors. Research indicates that hsa-miR-143-3p has potential as a cancer biomarker, significantly affecting tumor cell proliferation and metastasis while being closely associated with the regulation of various cellular processes, including apoptosis [28, 47, 48]. Its crucial role in cellular signaling involves multiple pathways, such as the PI3K/Akt, Wnt/β-catenin, Akt/STAT3, and Ras-Raf-MEK-ERK pathways, all of which contribute to the biological processes of tumors [28, 49]. hsa-miR-10a-5p functions as an oncogene in several cancers, promoting cell proliferation and metastasis [50]. In our study, the overexpression of hsa-miR-10a-5p in *C. sinensis* ^+^ HCC samples aligns with previous research in pancreatic cancer (PDAC), where it was identified as a key mediator of metastasis formation and was also shown by Fei et al. to promote pancreatic cancer growth [29, 50, 51]. hsa-miR-100-5p plays a crucial role in cancer progression by regulating apoptosis and cell growth, thereby influencing tumor development [30–32]. Our research findings are consistent with those of PeiJie Chen et al., who reported that the oncogenic hsa-miR-100-5p is associated with cellular viability, migration, and apoptosis in renal cell carcinoma (RCC) and is significantly upregulated in RCC tissues compared with normal adjacent tissue samples [52]. Furthermore, the expression variations of hsa-miR-100-5p in various cancers position it as a potential biomarker with promising applications in cancer diagnosis and prognosis assessment [52, 53]. Consistent with our findings, Tuba Gunel et al. discovered that the loss of hsa-let-7d-3p expression is associated with tumorigenesis, invasion, and metastasis in ovarian cancer [54, 55]. Ye Yang et al. found that the deregulation of certain miRNAs, such as hsa-let-7i-5p, in lung squamous cell carcinoma (SCC) suggests their involvement in the development and progression of the disease [56]. Among the miRNAs frequently dysregulated in cancer, hsa-miR-221 is considered of great importance [57]. hsa-miR-221-3p is commonly involved in regulating tumor-related biological processes, including influencing tumor cell growth by modulating metabolic pathways, while also playing an important role in cell proliferation, migration, and invasion [58]. Our study found that the downregulation of hsa-miR-221-3p in *C. sinensis* ^+^ HCC is closely associated with the metabolic regulatory functions of its target genes, suggesting that it may influence tumor cells by inhibiting metabolic pathways. Similarly, research by Chang-Jiang Shao et al. indicates that the depletion of hsa-miR-221-3p upregulates ferroptosis in gastric cancer cells through the upregulation of ATF3, which mediates the transcriptional inhibition of GPX4 and HRD1 [59]. On the basis of the findings of our study, the distinct expression profiles of miRNAs may contribute to the occurrence and progression of HCC. This underscores the importance of understanding miRNAs dysregulation in the context of *C. sinensis* infection and its potential implications for HCC pathogenesis.

During chronic liver injury, whether induced by viral or parasitic infections, drug toxicity, or alcohol abuse, both hepatic metabolic functions and the immune microenvironment become dysregulated [60, 61]. This disruption in systemic and local immune regulation results in chronic inflammation and fibrosis, ultimately promoting the development of HCC [62]. One of the most significant findings of our study was the identification of differentially expressed miRNAs between *C. sinensis*^+^ and *C. sinensis*^−^ HCC tumors. Similarly, Chao Yan et al. and Su Han et al. also demonstrated that miRNAs were differentially expressed in the liver of animals infected by *C. sinensis* [63, 64]. Furthermore, both our study and the mouse model described by Chao Yan et al. revealed consistent differential expression patterns for 14 miRNAs [63]. GO enrichment analysis revealed that many upregulated miRNAs are involved in immune system regulation. These findings are consistent with observations from animal models, where *C. sinensis*-derived miRNAs were shown to influence host immune responses significantly and pathophysiology; For instance, a recent study reported that Csi-let-7a-5p could activate M1-like macrophages, thereby exacerbating biliary inflammation and injury induced by *C. sinensis* [65]. In this study, we observed that miRNAs in *C. sinensis*^+^ HCC tumors are predominantly linked to metabolism, development, and cell cycle regulation. Recent studies have shown that metabolic reprogramming is a hallmark of tumor progression and resistance to therapy [45]. The downregulation of specific miRNAs may promote the metabolic flexibility of HCC cells, enabling them to adapt to the altered metabolic environment created by *C. sinensis* infection. Notably, our study also observed significant downregulation of let-7d-3p in human liver tissues, consistent with findings from Chao Yan et al. and Su Han et al. in mouse models of *C. sinensis* infection [63, 64]. These cross-species consistencies suggest that let-7d-3p may play a conserved and critical regulatory role in *C. sinensis* infection and its related pathological processes. This finding not only provides new insights into the pathological mechanisms of liver cancer induced by *C. sinensis* infection but also lays a solid foundation for developing targeted therapeutic strategies aimed at let-7d-3p. However, these studies have largely focused on nontumor liver tissues of animals. In contrast, our study is distinct in human HCC tissues associated with *C. sinensis* infection. Specifically, we emphasize miRNA alterations during cancer progression and use ceRNA network analysis to reveal complex molecular interactions. The dysregulation of miRNAs in *C. sinensis*^+^ HCC plays a pivotal role in shaping the immune TME and driving metabolic reprogramming, both of which may contribute to tumor progression. This dual impact on both immune and metabolic landscapes may provide a mechanistic basis for the enhanced progression of HCC in *C. sinensis*-infected patients.

Through the construction of the ceRNA network, we revealed complex interactions between lncRNAs, miRNAs, and mRNAs in *C. sinensis*^+^ HCC samples. We identified 149 lncRNAs, 8 miRNAs, and 66 mRNAs involved, which may potentially regulate the *C. sinensis* effect in HCC. This highlights the significant role of lncRNAs as sponges for miRNAs, regulating their availability to target mRNAs. The ceRNA network, therefore, provides a comprehensive view of how *C. sinensis* infection disrupts normal gene expression in HCC. These findings suggest that the key nodes in the ceRNA network could serve as biomarkers for disease progression and potential therapeutic targets. By targeting these critical regulatory points, it may be possible to reverse the oncogenic effects of miRNAs dysregulation. Additionally, the lncRNA–miRNA–mRNA axis could be exploited for the development of personalized therapies tailored to the molecular profile of patients with HCC who are infected with *C. sinensis*.

Nonetheless, our study has a few limitations. First, the relatively small sample size may limit the generalizability of our findings. We plan to expand the sample size to validate our current findings, strengthen their statistical power, and improve their generalizability. Second, similar differential expressions have been confirmed in related mouse models, supporting the validity of our bioinformatics findings. However, further experimental validation is required to confirm the functional significance of the predicted lncRNA-miRNA-mRNA interactions. Addressing these limitations in future studies will strengthen the robustness and clinical applicability of our findings.

## Conclusion

In summary, our study provides new insights by which *C. sinensis* infection promotes HCC progression through the dysregulation of miRNAs and the disruption of key biological processes such as immune regulation, and metabolism. The identification of lncRNA-miRNA-mRNA ceRNA networks highlights potential therapeutic targets that could lead to personalized treatment strategies for *C. sinensis* ^+^ HCC patients. Future research should focus on the functional validation of these targets and the development of novel therapies that exploit miRNAs dysregulation.

## Supplementary Information

Supplementary Material 1.

Supplementary Material 2.

Supplementary Material 3.

Supplementary Material 4.

## Data Availability

The raw sequencing data generated in this study has been deposited at SRA (PRJNA1168197).
