# Supplementary figures and images for: The microRNA landscape and regulatory network in Clonorchis sinensis-infected hepatocellular carcinoma: implications for tumor progression
Source: Parasit Vectors. 2025 Feb 21;18:68. doi: 10.1186/s13071-025-06689-z (PMC11846337; doi:10.1186/s13071-025-06689-z)

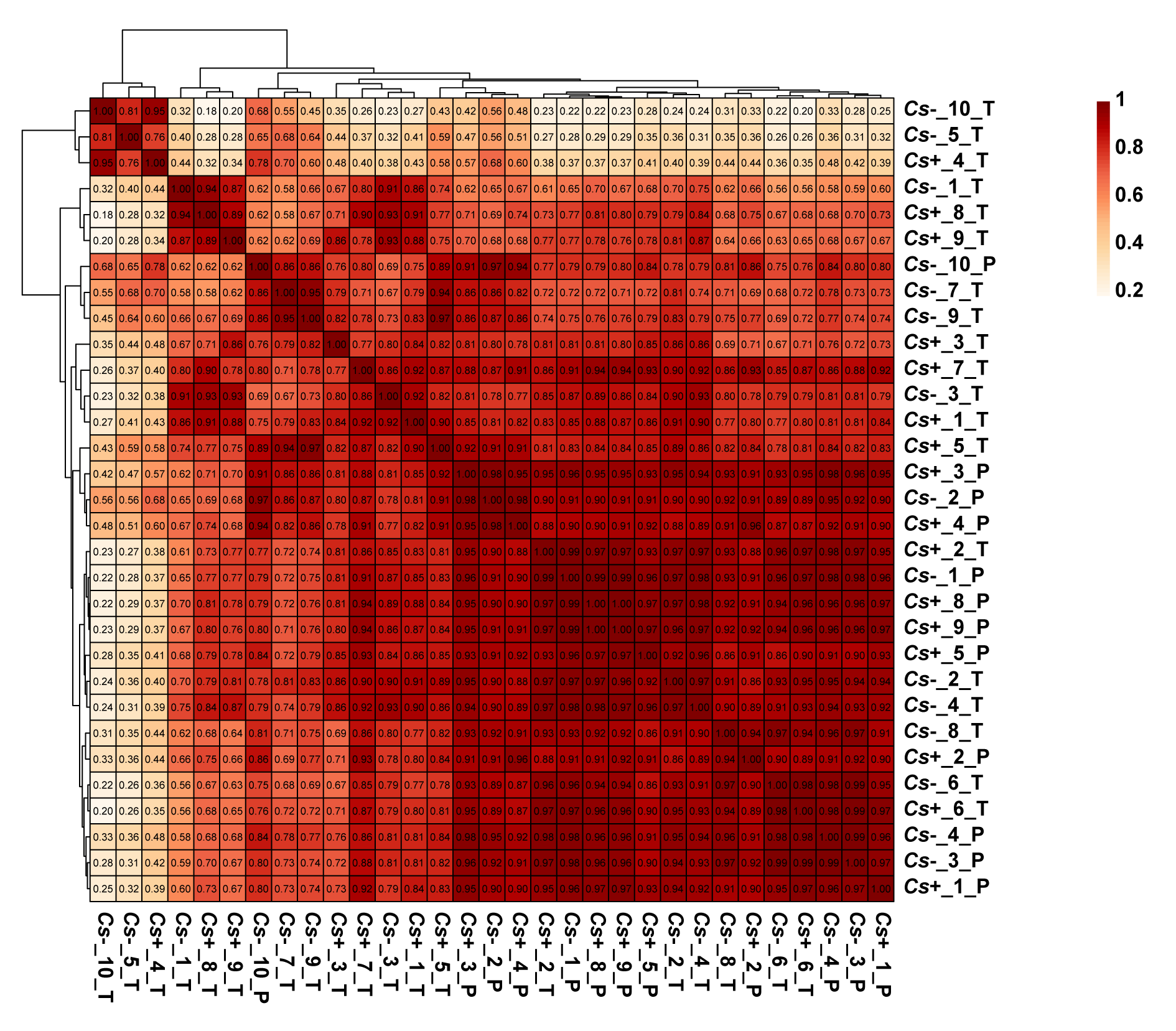

Supplement: Supplementary file 1 — Supplementary Material 1. [file 13071_2025_6689_MOESM1_ESM.tif]

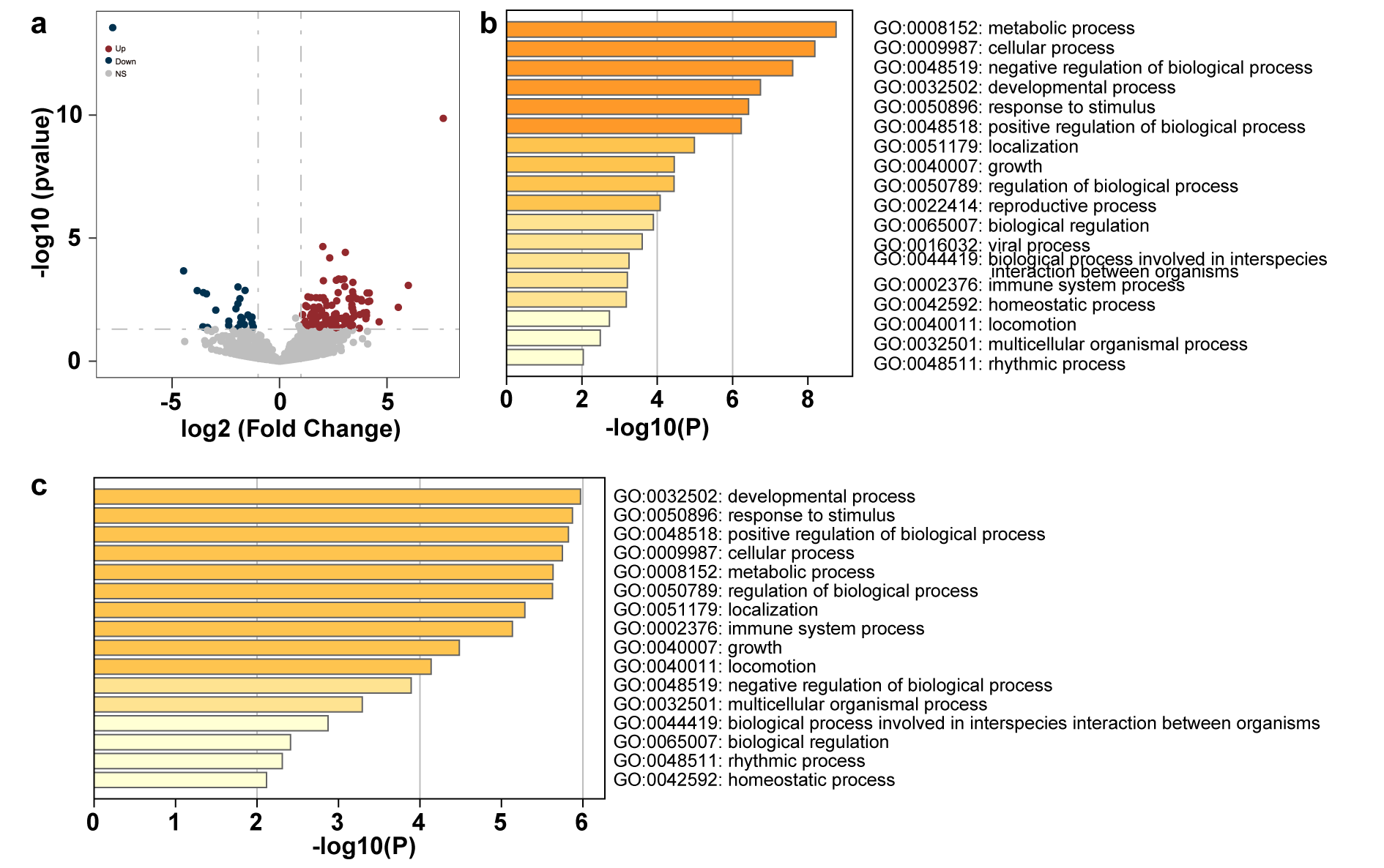

Supplement: Supplementary file 2 — Supplementary Material 2. [file 13071_2025_6689_MOESM2_ESM.tif]

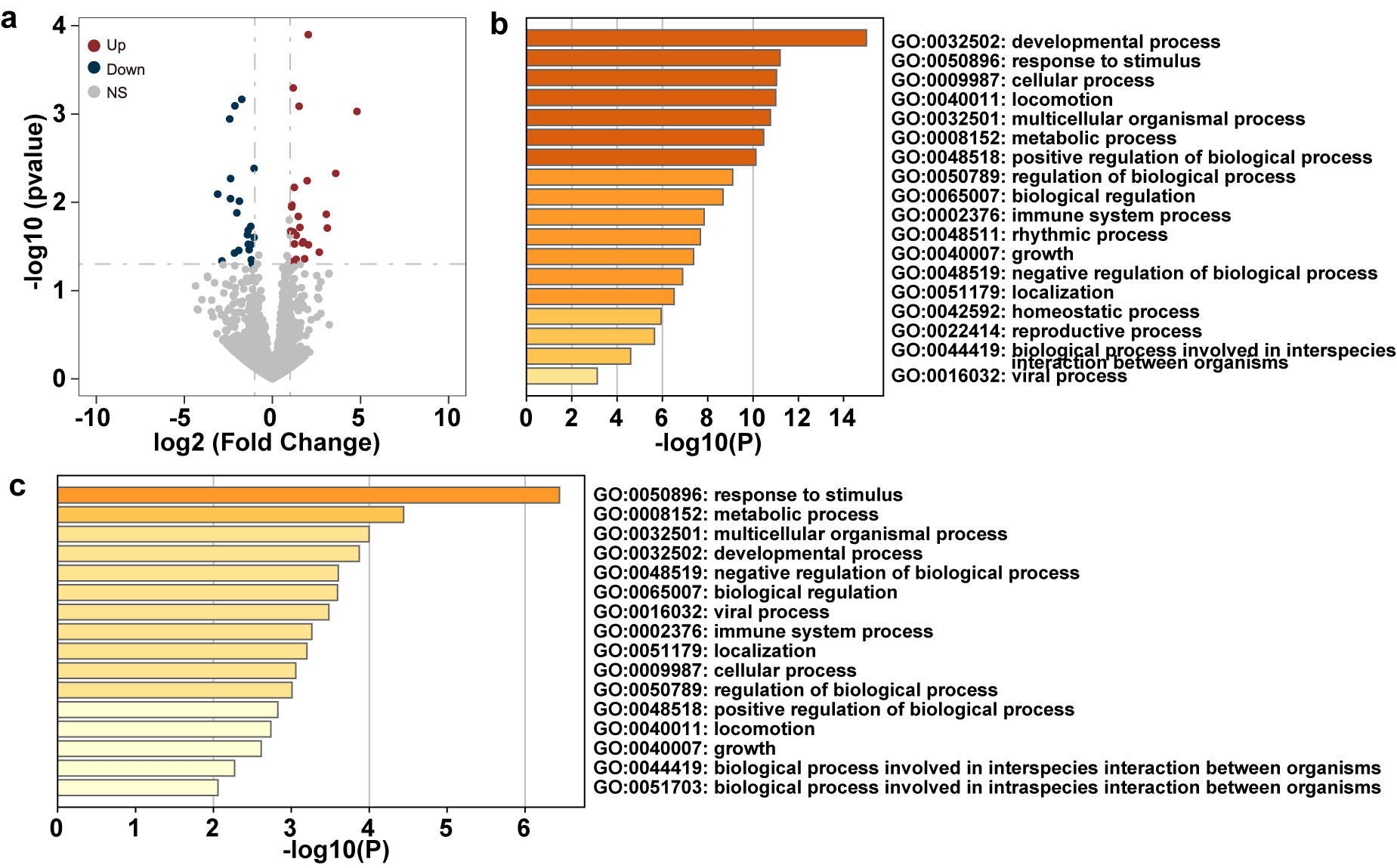

Supplement: Supplementary file 3 — Supplementary Material 3. [file 13071_2025_6689_MOESM3_ESM.tif]
